# Gear Fault Diagnosis Method Based on Multi-Sensor Information Fusion and VGG

**DOI:** 10.3390/e24111618

**Published:** 2022-11-06

**Authors:** Dongyue Huo, Yuyun Kang, Baiyang Wang, Guifang Feng, Jiawei Zhang, Hongrui Zhang

**Affiliations:** 1 School of Information Science and Engineering, Linyi University, Linyi 276000, China; 2 School of Logistics, Linyi University, Linyi 276000, China; 3 School of Life Science, Linyi University, Linyi 276000, China; 4 International College, Philippine Christian University, Manila 1004, Philippines; 5 Linyi Trade Logistics Science and Technology Industry Research Institute, Linyi 276000, China; 6 School of Mechanical and Vehicle Engineering, Linyi University, Linyi 276000, China

**Keywords:** gear fault diagnosis, multi-sensor information fusion, VGG

## Abstract

The gearbox is an important component in the mechanical transmission system and plays a key role in aerospace, wind power and other fields. Gear failure is one of the main causes of gearbox failure, and therefore it is very important to accurately diagnose the type of gear failure under different operating conditions. Aiming at the problem that it is difficult to effectively identify the fault types of gears using traditional methods under complex and changeable working conditions, a fault diagnosis method based on multi-sensor information fusion and Visual Geometry Group (VGG) is proposed. First, the power spectral density is calculated with the raw frequency domain signal collected by multiple sensors before being transformed into a power spectral density energy map after information fusion. Second, the obtained energy map is combined with VGG to obtain the fault diagnosis model of the gear. Finally, two datasets are used to verify the effectiveness and generalization ability of the method. The experimental results show that the accuracy of the method can reach 100% at most on both datasets.

## 1. Introduction

The gearbox is an important component in a mechanical transmission system and plays a key role in aerospace, wind power and other fields. Because of the complex structure of the gearbox and its continuous operation in harsh environments, it is easy to cause failures [1]. Gear failure is one of the main reasons for gearbox failure. Due to the complex working conditions of the gear and the easily affected tooth surface, its performance gradually degrades. When the gears are degraded to a certain extent, serious failures occur, resulting in huge economic losses and even major safety accidents [2,3]. Therefore, the intelligent diagnosis of gear faults is important research [4].

The traditional intelligent diagnosis method is mainly divided into three steps. First, the signal data of the sensor is collected when the machine is running. Second, feature extraction and selection are performed. Finally, the extracted features are fed into the model for training and fault classification. Many researchers have done a lot of research on gear fault diagnosis using traditional intelligent diagnosis methods [5,6,7,8,9,10]. Inturi et al. [11] extracted effective features of gear fault signals using DWT and EMD and input the extracted features into the SVM model for training. After completing several experiments, the accuracy rate obtained by combining SVM and DWT was better than that obtained by combining SVM and EMD. This is due to the problem of modal aliasing in EMD analysis compared to DWT. Therefore, the diagnostic accuracy obtained by the combination of DWT and SVM is higher. Wang et al. [12] first used the local weighted scatter smoothing method (LOWESS) to preprocess the signal spectrum, used WPT to extract signal features second and finally used the least squares support vector machine optimized by adaptive particle swarm optimization to diagnose fault types. After simulation experiments, the fault diagnosis was accurate. In the data preprocessing stage, the combination of LOWESS and WPT reduced the interference of noise to a certain extent, while enhancing the ability of certain features in classification. In the classification and diagnosis stage, the ASPO algorithm was used to select and adjust the optimal value of the parameters, which achieved higher accuracy than the traditional SVM algorithm. The rate reached 98.33%. Ramtek et al. [13] used Flexible Analytic Wavelet Transform (FAWT) to decompose signal data into sub-band signals and used various entropies to extract features from all sub-band signals, then input these features into a LS-SVM classifier. Since the feature of the log energy entropy has properties better than other entropies, it provided the best diagnostic accuracy for signal classes. The results of simulation experiments showed that the accuracy of this method is higher than that of Continuous Wavelet Transform (CWT), Discrete Wavelet Transformation (DWT), Wavelet Packet Transform (WPT), Dual-Tree Complex Wavelet Transform (DTCWT) and Tunable Q-factor Wavelet Transform (TQWT). Vos et al. [14] combined LSTM and SVM for diagnosing gear faults. Two-step LSTM and SVM were used for bearing fault diagnosis. Simulation experiments showed that the proposed method is more reliable and effective.

With the continuous development of modern industry, the complexity of machinery and equipment continues to increase, and the operating conditions of many gearboxes are usually variable operating conditions of speed and load. At the same time, the data generated by mechanical equipment have also grown exponentially, and mechanical fault diagnosis has entered the era of big data. Although traditional machine learning methods such as SVM have good performance on small sample datasets, these methods require a lot of expertise and domain knowledge, and the feature engineering steps need to be done manually. Since deep learning does not require manual feature extraction, deep learning has been applied to gear fault diagnosis by scholars [15,16,17,18,19,20]. Nguyen et al. [21] used ANC and DNN to build a sensitive gear fault diagnosis model that can effectively and accurately diagnose the types of faults with different shaft speeds and have high classification accuracy. Cui et al. [22] proposed a semi-supervised fault diagnosis method, which first extracted features from vibration signals through unsupervised training, then used a small amount of labeled data for supervised fine-tuning for fault diagnosis. Accuracies of 99.42% and 91.97% can be achieved on the bearing dataset and gear datasets, respectively. Cao et al. [23] proposed an unsupervised domain-shared convolutional neural network for efficient fault diagnosis of machines from stable to time-varying speeds. The method analyzed the original vibration signal data of bearings and gears at different speeds. As demonstrated by their experiments, the method has higher diagnostic accuracy, faster convergence speed and better robustness than other methods. Wu et al. [24] adopted a transfer learning method based on A2ResNet-aCoral to solve the problem of small sample datasets. After experimental verification, the accuracy of this method on a small sample set is 92.71%.

At present, the traditional gear fault diagnosis mainly focuses on the signal of a single sensor [25,26,27]. Dong et al. [28] used adversarial denoising autoencoders as feature extractors and introduced joint ensemble and statistical alignment methods to process the deep features of samples to reduce the geometric and statistical differences between the source and target domains. This improved the accuracy of the fault diagnosis. Hasan et al. [29] proposed a fault diagnosis method based on acoustic emission signal-based acoustic spectrum imaging (ASI) combined with transfer learning. ASI converts the spectral component amplitude of the windowed time-domain acoustic emission signal into a spectrogram and uses the image to represent the characteristics of the acoustic emission signal. It then uses CNN for feature extraction for diagnosis. The accuracy of this method can reach 94.67%. Li et al. [30] combined the autoencoder and the aggregated neural network to build a fault diagnosis model and used four loss functions to extract the features of the fault information, finally achieving an accuracy of 94.89%. Although the research using only a single sensor has the advantages of simplicity and speed, the fault information it contains is limited, and it has poor performance in fault tolerance.

In order to solve the problem of lower diagnostic accuracy under variable working conditions for the signal characteristics of a single sensor, information fusion and VGG are used to solve the problem of gear fault diagnosis. A gear fault diagnosis method based on multi-sensor information fusion and VGG is proposed. The main contributions of the paper are as follows:

1. The signal drawn directly using the plot function is no longer used as the input of the neural network. Instead, the power spectrum density energy diagrams obtained after power spectral density analysis of the original signal is used as the input of the neural network.
2. In order to obtain richer information and features, it is no longer only using the data collected by a single sensor for training and diagnosis. Instead, it uses data signals from multiple sensors, which are fused together and used for training and diagnostics.
3. In order to improve the accuracy and generalization ability of fault diagnosis, instead of using traditional machine learning methods, the obtained power spectrum density energy diagrams are combined with VGG16. Finally, its accuracy and generalization ability are verified on two datasets.

## 2. Methods

### 2.1. Convolutional Neural Network (CNN)

CNN is a deep neural network with a convolutional structure. It has powerful automatic feature extraction capabilities. The input layer, convolution layer, activation layer, pooling layer and fully connected layer constitute CNN. Convolution, activation and pooling operations can be reused. The convolution layer realizes the extraction of data features before the pooling layer reduces the dimensionality of the data and combines the extracted features through the fully connected layer to achieve classification.

The process of extracting features in the convolutional layer is the process of sliding the convolution kernel according to the specified step size and performing the convolution operation. The calculation process of the convolution operation is shown by Formula (1):

(1) $$X_{i+1}=W_{i}\;⊗\;X_{i}+b_{i},$$

where $X_{i}$ is the images input to the convolutional layer. $X_{i+1}$ represents the image after the convolution operation. The symbol ⊗ represents the convolution operation. $W_{i}$ represents the weight of the convolution kernel, and $b_{i}$ is the bias.

The activation layer is used to nonlinearly transform the feature map after the convolution operation through the activation function to improve the expression ability of the model [31]. The mathematical expression after adding the activation function is shown in Formula (2):

(2) $$y_{i}=f\left(x_{i+1}\right)=f\left(W_{i}\;⊗\;X_{i}+b_{i}\right),$$

where $y_{i}$ represents the feature map after the output of the activation layer. $f\left(*\right)$ represents the activation function, and the selection of different activation functions has different effects on the performance of the model.

The main purpose of the pooling layer is to reduce the dimensionality of the data, and the most used in CNN is Max Pooling. Its mathematical model is shown in Formula (3):

(3) $$f=Max\left(x_{m,n},x_{m+1,n},x_{m,n+1},x_{m+1,n+1}\right)$$

$$\left(1\leq m\leq M,1\leq n\leq N\right).$$

The fully connected layer synthesizes multiple features to be extracted. Its mathematical expression is shown in Formula (4):

(4) $$h_{\theta }\left(x_{i}\right)=\left[\begin{array}{c} p\left(y_{i}=1|x_{i};\theta \right)\\ p\left(y_{i}=2|x_{i};\theta \right)\\ \vdots \\ p(y_{i}=k|x_{i};\theta ) \end{array}\right]=\frac{1}{\sum_{j=1}^{k}e^{\theta_{j}^{T}x_{i}}}\left[\begin{array}{c} e^{\theta_{1}^{T}x_{i}}\\ e^{\theta_{2}^{T}x_{i}}\\ \vdots \\ e^{\theta_{k}^{T}x_{i}} \end{array}\right],$$

where $\theta_{1},\theta_{2},\ldots ,\theta_{k}$ are the learning parameters of the model, multiplied by $\frac{1}{\sum_{j=1}^{k}e^{\theta_{j}^{T}x_{i}}}$ to make the probability distribution between [0,1].

**Figure 1:**
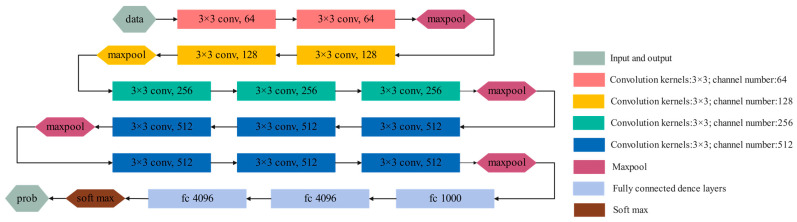
Network structure of VGG-16.

The VGG network proposed by the Visual Geometry Group of Oxford University is one of the current mainstream models of CNN [32]. To reduce the required parameters, the VGG network abandons the use of large-scale convolution kernels but uses multiple 3 × 3 convolution kernels. Under the premise of the same receptive field, the 5 × 5 convolution kernel is replaced by two 3 × 3 convolution kernels. The 7 × 7 convolution kernel is replaced by three 3 × 3 convolution kernels. The size of the pooling kernel is 2 × 2. Through two fully connected layers with 4000 nodes and an activation function, a 1 × 1 × 4096 vector is obtained. Then, through a fully connected layer of S (S is the number of categories) nodes, a 1 × 1 × S vector is obtained. Finally, the one-dimensional vector obtained through the fully connected layer is input to the softmax activation function, and the prediction result is converted into a probability distribution. Figure 1 shows the structure of VGG-16. The expression of the adopted ReLU activation function is shown in Formula (5). Therefore, VGG-16 has the advantages of simple structure, small convolution kernel, small pooling kernel, deeper layers and wider feature maps. At the same time, VGG-16 uses the ReLu function instead of Tanh and Sigmoid as the activation function because it is an unsaturated function, which can reduce the error of back propagation, and the speed of network convergence will be faster. This can greatly reduce training time.

(5) $$f\left(x\right)=\left\{\begin{array}{c} x,\;\;\;\;\;x\geq 0\\ 0,\;\;\;\;\;x<0 \end{array}\right.$$

VGG-16 was originally used for projects with 1000 categories, that is, N = 1000, because the fault category of gear fault diagnosis is much less than 1000. To improve the efficiency of the model, the number of nodes in the first two fully connected layers is halved. In other words, 4096 nodes of the first two fully connected layers are modified to 2048. The number of nodes of the last fully connected layer is consistent with the number of failure categories of the dataset.

### 2.2. Power Spectrum Density Energy Diagrams (PSDED)

Power spectral density (PSD) is a method to calculate the power of each frequency band of a signal. The definition of the PSD of a signal $x\left(n\right)$ is shown in Formula (6). The specific process of calculating the PSD of the signal is: first perform fast Fourier transform (FFT) on the signal $u\left(n\right)=0,1,\ldots ,N-1$, and then pass the spectral power of each frequency band through the absolute value form table out.

(6) $$P\left(\omega \right)=\sum_{k=-\infty }^{+\infty }r\left(k\right)e^{-i\omega k}$$

The formula for discrete Fourier transform is shown in Formula (7). Among them, $W_{N}=e^{-j2\pi /N},\;k=0,1,\ldots ,N-1$. Both $W_{N}$ and $U\left(k\right)$ have periodicity, and their period size is *N*.

(7) $$U\left(k\right)=\sum_{n=0}^{N-1}u\left(n\right)W_{N}^{kn}$$

**Figure 2:**
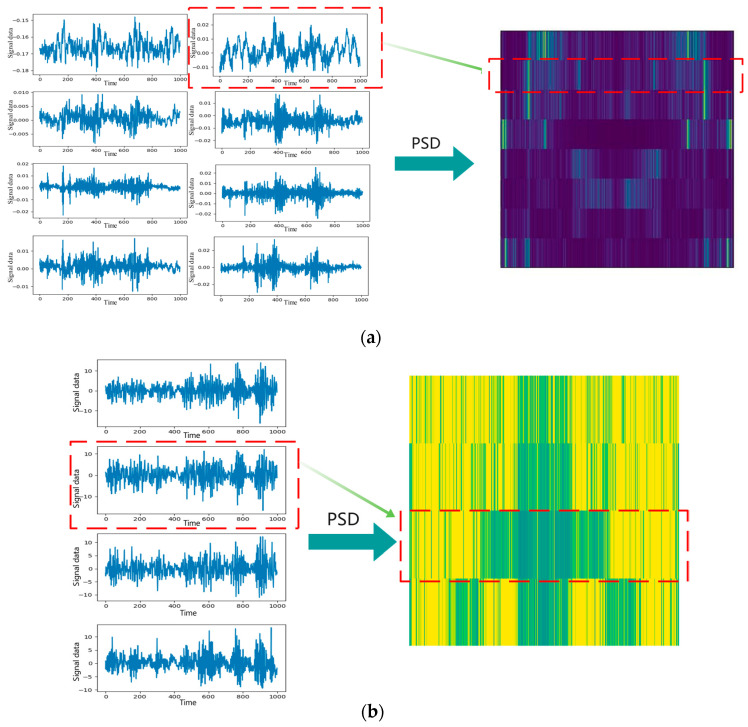
Power Spectrum Density Energy Diagrams: (**a**) 8 channels; (**b**) 4 channels.

The effect diagram obtained after PSD analysis is shown in Figure 2, where (a) is the PSDED with 8 channels and (b) is the PSDED with 4 channels.

### 2.3. Model Training and Diagnosis Process

**Figure 3:**
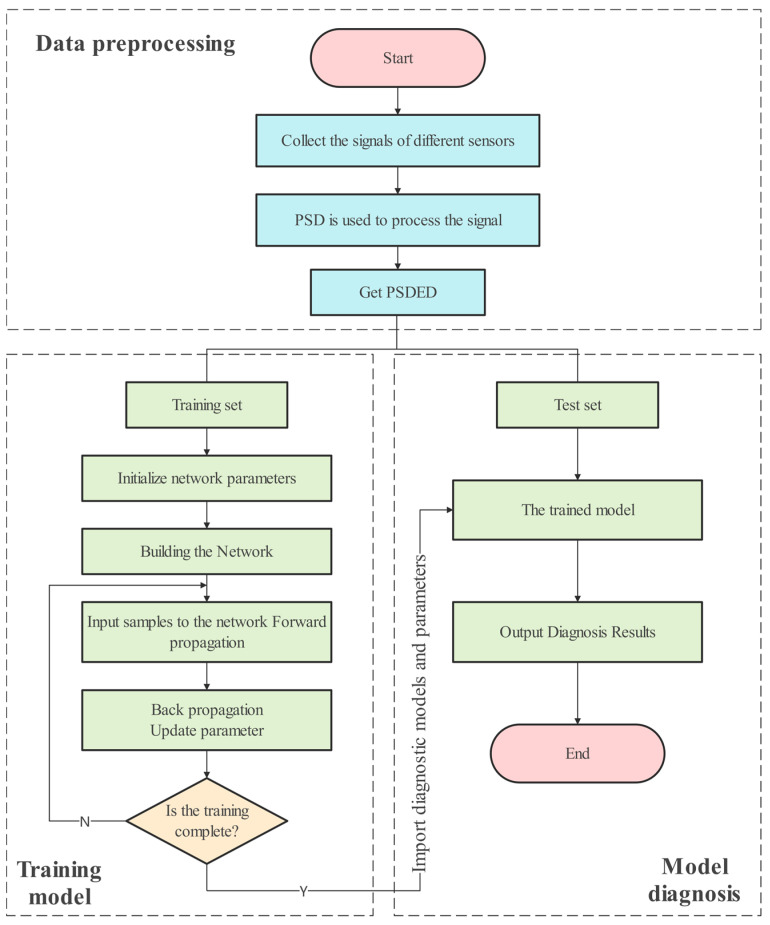
Flow chart of gear fault diagnosis.

Figure 3 shows a flowchart of the method. The process consists of three steps: data preprocessing, model training, and model diagnosis. The specific operation steps are as follows.

Step one: Data preprocessing. The PSD is calculated for each sensor vibration signal collected, and after PSD analysis, the information of multiple sensors is fused to obtain a two-dimensional picture, namely PSDED. A PSDED is a sample. The samples are divided into multiple data sets according to the working conditions, and the data sets are divided into training sets and test sets according to a certain proportion.

Step two: Train the model. Build the VGG model and initialize parameters. Input the divided dataset into the built model and train it. Finally, the trained model is obtained.

Step three: Model diagnosis. Test samples are fed into the trained model. The model will perform adaptive feature extraction and pattern recognition on the gear fault data. The final output of the diagnosis results.

## 3. Dataset

Two public gear failure datasets are used to verify the effectiveness and generalization ability of the proposed method. Dataset 1 is a five-category dataset of 8 sensors from Southeast University. Dataset 2 is a binary public dataset with four sensor data.

### 3.1. Dataset 1 from Southeast University

**Figure 4:**
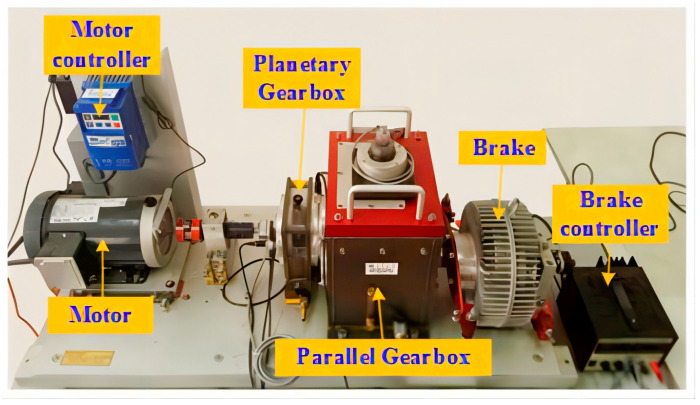
Experimental setup for gearbox dataset. Reprinted with permission from Ref. [33]. 2018, Siyu Shao.

Dataset 1 is a gearbox fault diagnosis dataset from Southeast University. These data were collected from Drivetrain Dynamic Simulator. The experimental setup of the gearbox dataset is shown in Figure 4 [33]. The dataset consists of a bearing dataset and a gear dataset. This paper selects the gear fault sub-dataset in this dataset. The data of the dataset were collected under two working conditions with the rotating speed system loads of “20hz and 0v” and “30hz and 2v”. The data set collected data of five types: health, crack at the tooth foot, lack of one tooth in the gear, crack at the root of the tooth foot and wear on the tooth surface. Each type of data is composed of signals collected by eight sensors. One means motor vibration. Two, three and four represent the vibration of the planetary gearbox in the three directions of X, Y and Z, respectively. Five represents the motor torque. Six and seven denote parallel gearboxes, respectively. Eight represents the three directions of vibration X, Y and Z. Table 1 describes the category information of the dataset.

The raw data collected under each condition (C = [‘20hz and 0v’, ‘30hz and 2v’]) and under each type were divided into D = 2000 segments. The dataset is treated as a five-category (T = [‘Health’, ‘Chipped’, ‘Miss’, ‘Root’, ‘Surface’]) fault diagnosis under two conditions. There are 2000 sample points for each category, and the total number of sample points is shown in Formula (8).

(8) $$D_{Total}=len\left(C\right)\times len\left(T\right)\times D$$

### 3.2. Dataset 2 from a Public Dataset

Dataset 2 is a gear fault diagnosis dataset disclosed in [34]. The dataset includes vibration measurements on healthy and damaged gearboxes under various loads (0–90%, in 10% steps) and a constant rotational speed of 30 Hz. Measurements were recorded with Spectra Quest’s Transmission Troubleshooting Simulator. The dataset is divided into two categories: health and damage. The signal data in each case were composed of arrays of signals measured separately by four sensors placed in different locations. The type description of this dataset is shown in Table 2.

The raw data collected under each condition (C = [‘Health’, ‘Broken’]) and each load (F = [‘0’, ‘10’, …, ‘90’]) were divided into D = 880; each segment has MD = 1000 data points, and each segment contains S = 4 time series measured by sensors placed at different locations. The total number of segments is as shown in Formula (9).

(9) $$D_{Total}=len\left(C\right)\times len\left(F\right)\times D$$

## 4. Experiment and Analysis

Several experiments were conducted on a Lenovo PC with Windows 10 64-bit operating system, Nvidia GeForce RTX 3060 GPU and 16 GB RAM. The operating environment of the experiment was: python3.9, Keras deep learning library implementation, cuda computing platform.

Each dataset was divided into a training set and a test set according to the ratio of 1:9. The batch size was set to 32. The number of iterations for training was set to 50. The learning rate was set to 0.0001.

### 4.1. Evaluation Indicators

Deep learning evaluation indicators were used to evaluate the performance of the deep learning model and were an important basis for model design. It is very limiting for evaluating models to count only the number of correct or incorrect predictions. Therefore, in order to verify the effectiveness of the proposed method from multiple angles and aspects, three evaluation indicators were used: loss function and accuracy, confusion matrix and t-distributed stochastic neighbor embedding (t-SNE).

#### 4.1.1. Loss Function and Accuracy

Cross-entropy describes the distance between two probability distributions. The smaller the cross-entropy, the closer the two probability distributions are. The cross-entropy function is defined as Formula (10), where n is the number of samples, m represents the number of categories, $y_{ic}$ is the label value and $p_{ic}$ is the predicted value.

(10) $$Loss=\frac{1}{n}\sum_{i}\sum_{c=1}^{m}y_{ic}log\left(p_{ic}\right)$$

In order to define the evaluation index accuracy (*Accuracy*), four concepts of True Positive, False Positive, True Negative and False Negative were proposed. *TP* (True Positive): The predicted value of the sample is consistent with the true value, and both are positive. *FP* (False Positive): The predicted value of the sample is positive, and the true value is negative. *FN* (False Negative): The predicted value of the sample is negative, and the true value is positive. *TN* (True Negative): The predicted value of the sample is consistent with the true value, and both are negative. The calculation formula of Accuracy is shown in Formula (11).

(11) $$Accuracy=\frac{TP+TN}{TP+TN+FP+FN}$$

#### 4.1.2. Confusion Matrix

**Figure 5:**
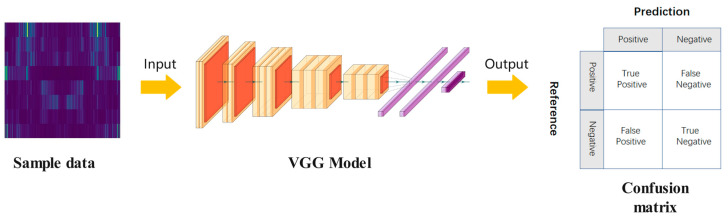
Structure of the confusion matrix.

A confusion matrix is a table of data used to understand the performance of a classification model. It helps us understand how the test data are divided into different classes. Each column of the confusion matrix represents the predicted class, and the total in each column represents the amount of data predicted for that class. Each row of the confusion matrix represents the true category of the data, and the total amount of data in each row represents the number of data instances of that category. The values in each column represent the amount of true data predicted to be in that category. The structure of the confusion matrix is shown in Figure 5.

#### 4.1.3. t-SNE

t-SNE is a common clustering method used for dimensionality reduction and cluster visualization of numerical variables. The main idea is to use the conditional probability to represent the similarity of the distance of high-dimensional distribution points. A cluster generated by clustering is a collection of data objects that are similar to objects in the same cluster and different from objects in other clusters. t-SNE mainly consists of two steps:

Step 1: a probability distribution between high-dimensional objects is constructed by t-SNE, so that similar objects have a higher probability of being selected, and dissimilar objects have a lower probability of being selected. Step 2: the probability distribution of these points in the low-dimensional space is constructed by t-SNE, so that the two probability distributions are as similar as possible.

### 4.2. Results and Analysis of Dataset 1

Five types of data were collected under two working conditions in the dataset. The power spectral density of all collected signals were calculated and analyzed, and the power spectral density energy map (PSDED) was obtained. The dataset was divided into a training set and a test set by a 9:1 ratio. The sample data are shown in Table 3.

**Figure 6:**
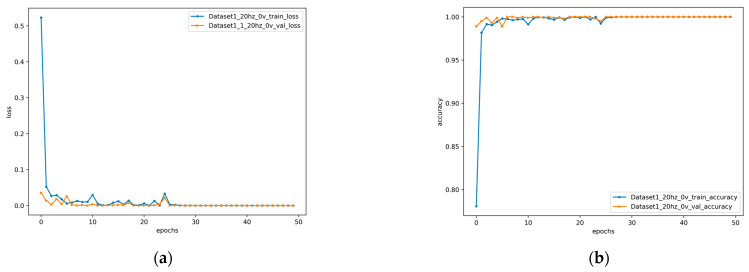
Loss and accuracy of 20hz0v: (**a**) loss; (**b**) accuracy.

**Figure 7:**
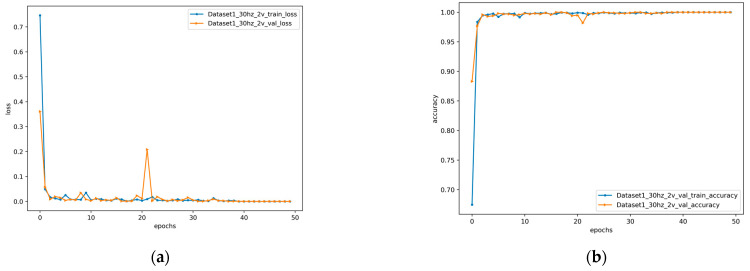
Loss and accuracy of 30hz2v: (**a**) loss; (**b**) accuracy.

The proposed method was used to diagnose gear faults in the case of 20hz0v and 30hz2v. The loss value and accuracy obtained from the training set and test set in the two cases are shown in Figure 6 and Figure 7. It can be seen from the figure that the proposed method achieved low loss value and high accuracy on both the training set and the test set under two variable working conditions. At the same time, it also had a good performance in the convergence speed. The loss value and accuracy obtained on the training set and test set are shown in Table 4. The proposed method achieved 100% accuracy under both variable conditions. Under the condition of 20hz0v, the loss of 1.54 × 10^−7^ and the accuracy of 100% were obtained on the training set, and the loss of 3.6 × 10^−10^ and the accuracy of 100% were obtained on the validation set. Under the condition of 30hz2v, a loss of 1.75 × 10^−7^ and an accuracy of 100% were achieved on the training set, and a loss of 0.00019 and an accuracy of 100% were achieved on the validation set.

**Figure 8:**
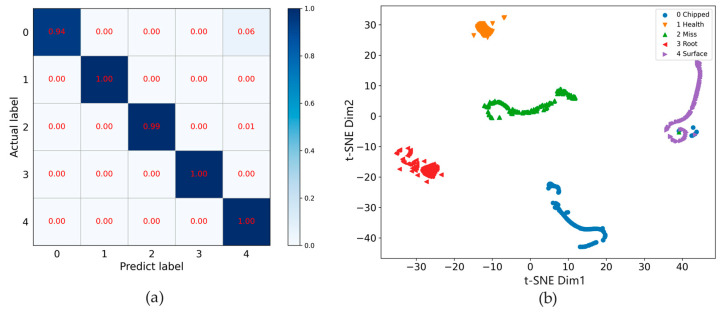
Confusion matrix and t-SNE of 20hz0v: (**a**) Confusion matrix; (**b**) t-SNE.

**Figure 9:**
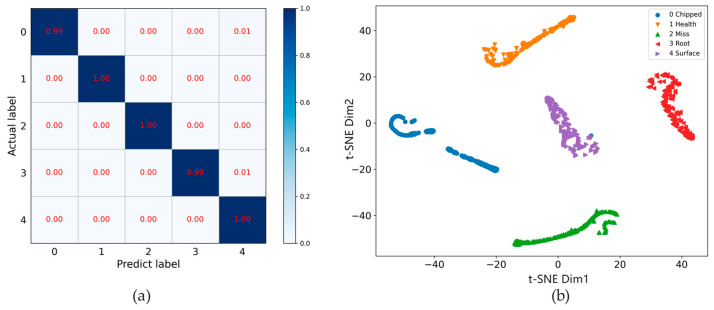
Confusion matrix and t-SNE of 30hz2v: (**a**) Confusion matrix; (**b**) t-SNE.

**Figure 10:**
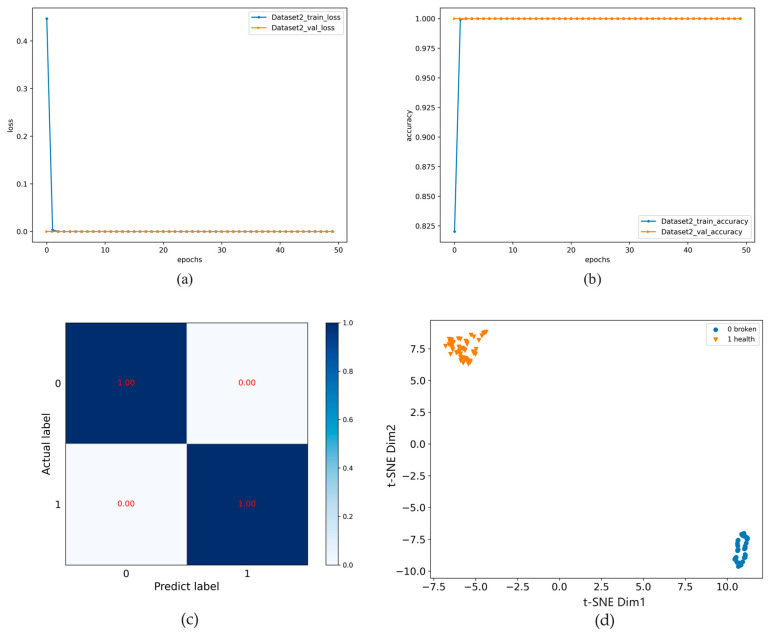
Results of Dataset 2: (**a**) loss; (**b**) accuracy; (**c**) Confusion matrix; (**d**) t-SNE.

In order to more intuitively show the effect of the proposed method in the fault diagnosis of variable working conditions, the confusion matrix and t-SNE of 20hz0v and 30hz2v are shown in Figure 8 and Figure 9, respectively. It can be seen from Figure 8 that under the working condition of 20hz0v, the accuracy of identifying the health status, Crack occurrence in the root of the gear feet and Wear occurrence in the surface of the gear reached 100%. The misclassification of the proposed method was mainly to identify 6% of Crack occurrence in the gear feet as Wear occurs in the surface of the gear and 1% of Missing one foot in the gear as Wear occurs in the surface of the gear. Correspondingly, it can be seen in the cluster diagram in Figure 8b that some points belonging to the 0th and 2nd categories were identified in the 4th category. It can be seen from Figure 10 that under the working condition of 30hz2v, the accuracy of identifying the health status, missing one foot in the gear and wear occurrence in the surface of gear reached 100%. The misclassification of the proposed method was mainly to identify 1% of Crack occurrences in the gear feet as Wear occurs in the surface of the gear, and 1% of Crack occurrences in the root of the gear feet as Wear occurs in the surface of the gear. Correspondingly, it can be seen in the cluster diagram in Figure 10b that some points belonging to the 0th and 3rd categories were identified in the 4th category.

In order to verify that multi-sensor information fusion can reflect the signal characteristics of gear health and damage more comprehensively and completely, each sensor used the same method to convert the signal into PSDED and input it as a sample to VGG for training. The accuracies of each sensor and multiple sensors are shown in Table 5. In the case of 20hz0v, the accuracy rate of using multi-sensor information fusion was as high as 100%, while the accuracy rate of using a single sensor varied from 95.6% to 99.6%, with an average accuracy rate of 98%. In the case of 30hz2v, the accuracy rate of using multi-sensor information fusion was 100%, the accuracy rate of using a single sensor was between 96.7% and 92.4% and the average accuracy rate was only 94.7%. Therefore, the use of multi-sensor information fusion can provide more comprehensive and accurate information for gear fault diagnosis, thereby improving the accuracy of fault identification.

To verify the effectiveness of the proposed method, a comparison between the experimental results and other papers is shown in Table 6. As can be seen from Table 6, the accuracy obtained by using the model multi-label recurrent translation adversarial network (MCTAN) [35] based on the multimodal neural network was about 68%. The accuracy obtained by using the multimodal neural-network-based model [36] was 97.83%. The accuracy obtained by using the wavelet transform and VGG16 [33] was 99.64%. This clearly shows that the proposed method can achieve better results.

### 4.3. Results and Analysis of Dataset 2

The dataset was divided into healthy and damaged categories under various loads and a constant rotational speed of 30 Hz. The power spectral density of the collected signal was calculated and analyzed to obtain a power spectral density energy map (PSDED). The dataset was divided into a training set and a test set in a ratio of 9:1. The sample data is shown in Table 7.

VGG neural network was used to train 50 epochs, and the training results are shown in Figure 10. As shown in Figure 10a,b, the loss value and accuracy of the model on the test set were 0 and 100%, respectively. The confusion matrix and t-SNE are shown in Figure 10c,d. The results show that the proposed method performed well on this dataset, reaching almost 100% accuracy. Therefore, the proposed method still has good generalization ability in the fault diagnosis of gears with different damage categories in different datasets.

To verify the effectiveness of the proposed method, the experimental results compared to other methods using dataset 2 are shown in Table 8. As seen in Table 8, the accuracies of the method “Long Short Term Memory (LSTM) and Particle Swarm Optimization (PSO)” [37] and the method “LSTM and Cuckoo” [37] were both 87.5%, and the accuracy of the method “Empirical Mode Decomposition (EMD) and Artificial Neural Network (ANN)” [34] was about 73%. It is clearly shown that the proposed method can achieve better results.

## 5. Conclusions

Aiming at the problem that it is difficult to effectively identify the fault types of gears with traditional methods under complex and changeable conditions, a fault diagnosis method based on information fusion and a convolutional neural network is proposed. First, the data power spectral density was calculated on the raw signals collected by multiple sensors to obtain the frequency domain signal, which was converted to a power spectral density energy map after information fusion and PSD analysis. Second, the obtained energy map was input to VGG16 as a sample for training and diagnosis. Then two datasets were used to verify the effectiveness and generalization ability of the proposed method. For dataset 1, the diagnostic accuracy under the two working conditions was obtained, which reached 100%. Then the accuracies of single-sensor and multiple-sensors were compared. Under the two working conditions, the accuracy of multi-sensor information fusion was 2% and 5.3% higher than that of a single-sensor, respectively. For dataset 2, its fault diagnosis rate was as high as 100%. Therefore, the fusion of the data of multiple sensors can provide more comprehensive and accurate information for gear fault diagnosis, and the combination with VGG16 can greatly improve the accuracy of fault identification. In future work, more advanced classifiers will be considered for gear fault diagnosis to improve its accuracy and robustness.

## Figures and Tables

**Table 1:** Description of the type in the dataset 1.

Type	Description
Chipped	Cracks in the gear feet
Health	Healthy gear
Miss	Missing one of feet in the gear
Root	Cracks at the root of the tooth
Surface	Wear on gear surfaces

**Table 2:** Description of the type in the dataset 2.

Type	Description
Broken	Broken gear
Health	Healthy gear

**Table 3:** Number of samples for Dataset 1.

Label	0	1	2	3	4
	Chipped	Health	Miss	Root	Surface
20hz_0v	1800	1800	1800	1800	1800
	200	200	200	200	200
30hz_2v	1800	1800	1800	1800	1800
	200	200	200	200	200

**Table 4:** Loss and accuracy of 20hz0v and 30hz2v.

	Train Loss	Train Acc	Val Loss	Val Acc
20hz_0v	1.54 × 10^−7^	1.0	3.6 × 10^−10^	1.0
30hz_2v	1.75 × 10^−7^	1.0	0.00019	1.0

**Table 5:** Accuracy of single-channel and multi-channel.

Channel	20hz_0v	30hz_2v
Sensor 1	99.4%	94.4%
Sensor 2	99.6%	95.9%
Sensor 3	95.9%	96.7%
Sensor 4	98.4%	96.2%
Sensor 5	98.1%	93.9%
Sensor 6	98.6%	96.6%
Sensor 7	95.6%	92.6%
Sensor 8	98.4%	92.4%
Fusion from sensor 1 to sensor 8	100%	100%

**Table 6:** Comparison with results from different papers using dataset 1.

Methods	Accuracy
MCTAN [35]	68%
multimodal neural-network-based model [36]	97.83%
wavelet transform and VGG16 [33]	99.64%
Proposed method	100%

**Table 7:** Number of samples for Dataset 2.

Label	0	1
	Broken	Health
Train	792	792
Test	88	88

**Table 8:** Results comparison of different methods about dataset 2.

Methods	Accuracy
EMD and ANN [34]	73%
“LSTM + PSO” and “LSTM + Cuckoo” [37]	87.5%
Proposed method	100%

## Data Availability

The datasets used in the study are available from the corresponding authors upon reasonable request.
